# Early versus deferred androgen suppression therapy for patients with lymph node-positive prostate cancer after local therapy with curative intent: a systematic review

**DOI:** 10.1186/1471-2407-13-131

**Published:** 2013-03-19

**Authors:** Frank Kunath, Bastian Keck, Gerta Rücker, Edith Motschall, Bernd Wullich, Gerd Antes, Joerg J Meerpohl

**Affiliations:** 1 Department of Urology, University Clinic Erlangen, Krankenhausstraße 12, 91054, Erlangen, Germany; 2 German Cochrane Centre, Institute of Medical Biometry & Medical Informatics, University Medical Centre Freiburg, Freiburg, Germany; 3 Institute of Medical Biometry and Medical Informatics, University Medical Centre Freiburg, Freiburg, Germany; 4 Pediatric Hematology and Oncology, Centre for Pediatrics & Adolescent Medicine, University Medical Centre Freiburg, Freiburg, Germany

**Keywords:** Prostatic neoplasms, Lymphatic metastasis, Lymph node excision, Androgen suppression therapy, Systematic review, Meta-analysis

## Abstract

**Background:**

There is currently no consensus regarding the optimal timing for androgen suppression therapy in patients with prostate cancer that have undergone local therapy with curative intent but are proven to have node-positive disease without signs of distant metastases at the time of local therapy. The objective of this systematic review was to determine the benefits and harms of early (at the time of local therapy) versus deferred (at the time of clinical disease progression) androgen suppression therapy for patients with node-positive prostate cancer after local therapy.

**Methods:**

The protocol was registered prospectively (CRD42011001221; http://www.crd.york.ac.uk/PROSPERO). We searched the MEDLINE, EMBASE, and CENTRAL databases, as well as reference lists, the abstracts of three major conferences, and three trial registers, to identify randomized controlled trials (search update 04/08/2012). Two authors independently screened the identified articles, assessed trial quality, and extracted data.

**Results:**

Four studies including 398 patients were identified for inclusion. Early androgen suppression therapy lead to a significant decrease in overall mortality (HR 0.62, 95% CI 0.46-0.84), cancer-specific mortality (HR 0.34, 95% CI 0.18-0.64), and clinical progression at 3 or 9 years (RR 0.29, 95% CI 0.16-0.52 at 3 years and RR 0.49, 95% CI 0.36-0.67 at 9 years). One study showed an increase of adverse effects with early androgen suppression therapy. All trials had substantial methodological limitations.

**Conclusions:**

The data available suggest an improvement in survival and delayed disease progression but increased adverse events for patients with node-positive prostate cancer after local therapy treated with early androgen suppression therapy versus deferred androgen suppression therapy. However, quality of data is low. Randomized controlled trials with blinding of outcome assessment, planned to determine the timing of androgen suppression therapy in node-positive prostate cancer using modern diagnostic imaging modalities, biochemical testing, and standardized follow-up schedules should be conducted to confirm these findings.

## Background

Prostate cancer is a relevant tumor with an increased morbidity and mortality [1,2]. The treatment options for prostate cancer confined to the prostate gland (localized disease) consist of radical prostatectomy or radiotherapy [3]. In men with low and intermediate risk localized prostate cancer (cT1a-T2b, Gleason score 2–7, PSA < 20 ng/mL) and life expectancy > 10 years [3], the goal of local therapy is the eradication of disease (local therapy with curative intent). The prognosis for these patients is excellent. However, advanced-stage prostate cancer with regional lymph node involvement or metastases is usually regarded as systematic disease with a potentially increased risk for morbidity and mortality.

Since the primary work of Huggins & Hodges on hormonal ablation therapy in prostate cancer [4], androgen suppression therapy (AST) has become an important non-curative therapeutic option to slow the progression of advanced prostate cancer [5]. The androgen testosterone is essential for prostate cell growth, and its suppression is therefore important in prostate cancer therapy. Androgen suppression can be achieved either by surgical castration or by luteinizing hormone releasing hormone (LHRH) agonists. LHRH agonists were found to be as effective as surgical castration with bilateral orchiectomy [5]. Antiandrogens inhibit the action of the circulating hormones at the level of androgen receptors in prostate cells. This therapy option is recommended for short-term administration in patients receiving LHRH agonists and non-steroidal antiandrogen monotherapy as an alternative to castration in patients with locally advanced prostate cancer [5]. LHRH antagonists are a new family of AST agents. However, whether they have advantages over LHRH agonists has not yet been determined [5].

Patients that have undergone local treatment for supposedly localized disease with curative intent but were proven to be node-positive due to a definitive pathological examination that revealed no distant metastases are a therapeutic challenge because of the controversy concerning when to initiate hormonal therapy [5]. For these asymptomatic patients, therapy options that further slow progression with potentially increased side effects must be carefully balanced with a “wait and see” attitude and the possibility that this approach will increase the rate of disease progression. Therefore, two different types of AST administration are usually discussed: administration at the time of local therapy (early AST) or administration when there are signs and symptoms of clinical disease progression (deferred AST) [5]. To date, no systematic review has critically assessed the benefits and harms of early versus deferred AST for the subgroup of patients presenting with lymph node-positive disease at the time of local therapy.

## Methods

The protocol was prospectively registered in the ‘International prospective register of systematic reviews’ (http://www.crd.york.ac.uk/PROSPERO;CRD42011001221). We conducted a combination of electronic and manual searches. First, we identified potentially eligible studies from the CENTRAL (Cochrane Library 2011/Issue 2), MEDLINE (Ovid, 1946-02/2011), and EMBASE (DIMDI, 1947-02/2011) databases. The search strategy was adapted for each electronic database (Table 1). Second, we screened reference lists and performed electronic searches for abstracts on the websites of major conferences (initial search 18/05/2011): the American Society of Clinical Oncology (ASCO, 2004-18/05/2011, http://jco.ascopubs.org), the European Association of Urology (EAU, 2004-18/05/2011, http://www.uroweb.org), and the American Urological Association (AUA ,2002-2007, http://www.abstracts2view.com/aua_archive/; 2008, http://www.abstracts2view.com/aua; 2009-18/05/2011, http://www.jurology.com). We also searched the following three trial registers for completed or ongoing studies (initial search 18/05/2011): Current Controlled Trials (ISRCTN, http://www.controlled-trials.com), ClinicalTrials.gov (http://www.clinicaltrials.gov), and the clinical trials search portal of the World Health Organization: (ICTRP, http://www.who.int/ictrp/en/). We updated our search on April 8, 2012.

**Table 1:** Search strategies

**Database**	**Search strategy**
Ovid MEDLINE® In-Process & Other Non-Indexed Citations, Ovid MEDLINE® Daily and OVID MEDLINE® (1946-April 8, 2012)	1: Prostatic Neoplasms/; 2: (prostat* adj3 (cancer* or tumo* or neoplas* or carcinom* or malign*)).tw.; 3: 1 or 2; 4: Lymph Nodes/pa, su; 5: Lymphatic Metastasis/; 6: Neoplasm Invasiveness/; 7: (nod* adj3 positiv*).mp.; 8: N1.mp.; 9: D1.mp.; 10: N2.mp.; 11: (lymph* adj3 (metastas* or tumo* or neoplas* or carcinom* or malign*)).mp.; 12: 4 or 5 or 6 or 7 or 8 or 9 or 10 or 11; 13: Lymph Node Excision/; 14: lymphadenectom*.mp.; 15: (lymph* adj3 (surg* or operat* or excis* or removal*)).mp.; 16: 13 or 14 or 15; 17: randomized controlled trial.pt.; 18: controlled clinical trial.pt.; 19: placebo.ab.; 20: drug therapy.fs.; 21: randomly.ab.; 22: trial.ab.; 23: groups.ab.; 24: randomized.ab.; 25: 17 or 18 or 19 or 20 or 21 or 22 or 23 or 24; 26: exp animals/ not humans.sh.; 27: 25 not 26; 28: 3 and 12 and 16 and 27
EMBASE (1947-April 8, 2012)	1: EM74; 2: CT=("PROSTATE TUMOR"; "PROSTATE CANCER"; "PROSTATE ADENOCARCINOMA"; "PROSTATE CARCINOMA"); 3: (prostat* and (cancer* or tumo* or neoplas* or carcinom* or malign*))/same sent; 4: 2 OR 3; 5: CT=("LYMPH NODE"; "MESENTERY LYMPH NODE"; "PARAAORTIC LYMPH NODE"; "PELVIS LYMPH NODE"); 6: CT="LYMPH NODE METASTASIS"; 7: CT="CANCER INVASION"; 8: (nod* and positiv*)/same sent; 9: N1 or N2 or D1; 10: (lymph* and (metasta* or tumo* or neoplas* or carcinom* or malign*))/same sent; 11: 5 OR 6 OR 7 OR 8 OR 9 OR 10; 12: CT=("LYMPHADENECTOMY"; "LYMPH NODE DISSECTION"; "PELVIS LYMPHADENECTOMY"); 13: (lymph* and (surg* or operat* or excis* or remov*))/same sent; 14: lymphadenectom*; 15: 12 OR 13 OR 14; 16: 4 AND 11 AND 15; 17: su=medline; 18: 16 not 17; 19: CT=("CONTROLLED CLINICAL TRIAL"; "RANDOMIZED CONTROLLED TRIAL"); 20: CT="RANDOMIZATION"; 21: CT="DOUBLE BLIND PROCEDURE"; 22: CT="SINGLE BLIND PROCEDURE"; 23: CT="PROSPECTIVE STUDY"; 24: RANDOM*; 25: ((SINGL* OR DOUBL*) AND (BLIND* OR MASK*))/SAME SENT; 26: (CONTROLLED AND TRIAL)/SAME SENT; 27: ti=trial; 28: groups; 29: 19 OR 20 OR 21 OR 22 OR 23 OR 24 OR 25 OR 26 OR 27 OR 28; 30: 18 AND 29
Cochrane Database of Systematic Reviews, Cochrane Central Register of Controlled Trials (CENTRAL)	1: MeSH descriptor Prostatic Neoplasms, this term only; 2: (prostat* NEAR/3 (cancer* OR tumo* OR neoplas* or carcinom* or malign*)); 3: (1 OR 2); 4: MeSH descriptor Lymph Nodes, this term only; 5: MeSH descriptor Lymphatic Metastasis, this term only; 6: MeSH descriptor Neoplasm Invasiveness, this term only; 7: (nod* NEAR/3 positiv*); 8: (N1 OR N2 OR D1); 9: (lymph NEAR/3 (metastas* OR tumo* OR neoplas* OR carcinom* OR malign*)); 10: (4 OR 5 OR 6 OR 7 OR 8 OR 9); 11: MeSH descriptor Lymph Node Excision, this term only; 12: (lymphadenectomy); 13: (lymph* NEAR/3 (surg* OR operat* OR excis* OR removal*)); 14: (11 OR 12 OR 13); 15: (3 AND 10 AND 14)

Date of last search: April 8, 2012; Responsible searcher: Kunath, Motschall.

We considered parallel group RCTs that met the following criteria: patients had (1) node-positive prostate cancer at the time of local therapy (radical prostatectomy with lymphadenectomy or radiotherapy with either mandatory imaging or histological lymph node assessment), (2) received no prior AST, and (3) had no signs of distant metastases at the time of study entry. (4) All of the studies compared early AST (initiated at the time of local therapy) with deferred AST (initiated at the time of disease progression) for the treatment of advanced prostate cancer and (5) reported data on overall survival, cancer-specific survival, progression-free survival, discontinuation due to adverse events, or any adverse events. Whether LHRH antagonists have advantages over LHRH agonists or antiandrogens has not yet been determined [5]. However, LHRH antagonists are not part of this systematic review. We excluded publications reporting on patients who developed lymph node metastasis after local treatment (by radical prostatectomy or radiotherapy). We did not impose any limitations based on the age or ethnicity of the participants or the language of the publication.

One author (FK) screened all of the titles and abstracts of the citations identified by our search strategy and only excluded citations that were clearly irrelevant or were retrieved from more than one database (exclusion of duplicate entries). As a next step, two review authors (FK, BK) independently examined the full-text reports, identified relevant studies, assessed the risk of bias (random sequence generation, allocation concealment, blinding, incomplete outcome data, selective reporting, and other sources of bias) and extracted data on study/patient characteristics as well as data on our predefined outcomes. Disagreements were resolved by consensus or, if necessary, through discussion with a third review author (JM).

For statistical data analysis, we used RevMan 5.1 software provided by The Cochrane Collaboration (http://www.cochrane.org). For time-to-event outcomes, we either extracted hazard ratios (HR) with their 95% confidence intervals (CI) or used an indirect estimation method for estimation [6-8] if HR were not given (see overall survival and cancer-specific survival). If this was not possible, we calculated risk ratios (RR) with their 95% CI at certain time points (see clinical progression at 3 or 9 years). We assessed statistical heterogeneity (Chi^2^, I^2^) and used a fixed-effect model for I^2^ < 50%. A random-effects model was used for sensitivity analysis if I^2^ > 50%.

## Results

### Search results

**Figure 1:**
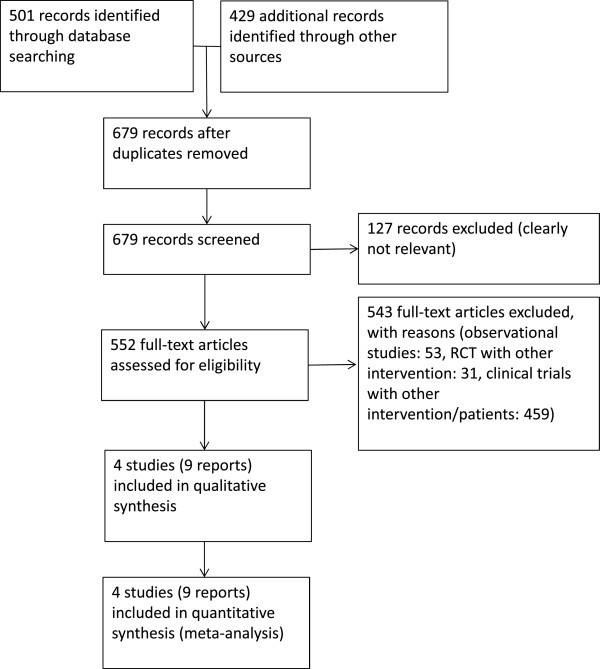
Search results.

The literature search identified a total of 930 citations. A total of 9 reports on 4 studies were finally included in the review. For details on results of the search, see Figure 1. We identified no ongoing studies, and no further subgroup analysis of excluded studies fulfilled our predefined inclusion criteria. We identified no additional study with our updated search. The following trials were included in our analysis: (1) RTOG-85-31, which was performed by the Radiation Therapy Oncology Group [9,10], (2) a study published by Granfors et al. [11,12], (3) EST-3886, a study performed by the Eastern Cooperative Oncology Group [13-15], and (4) EPC program, which was performed by the Early Prostate Cancer Program [16,17]. Lymph-node assessment by either imaging or surgical means was mandatory in all studies. Patients were stratified to nodal status before randomization. From three studies (RTOG-85-31, Granfors et al., EPC program), we included only the subgroups of patients with node-positive prostate cancer after local therapy with curative intent. Only one study (EST-3886) specified the total number of assessed lymph nodes as well as the number of positive lymph nodes in the lymphadenectomy specimen (Table 2). Table 3 reports details on the study characteristics, and Table 2 provides information on patients’ baseline characteristics. The quality of evidence was hampered by the risk of bias. For details, see Tables 4 and 5.

**Table 2:** Baseline patient characteristics

	**RTOG-85-31**[9,10]		**Granfors et al.**[11,12]		**EST-3886**[13-15]		**EPC program**[16,17]
	**early AST (n = 98)**	**deferred AST (n = 75)**	**early AST (n = 19)**	**deferred AST (n = 20)**	**early AST (n = 47)**	**deferred AST (n = 51)**	**early/deferred AST (n = 150) (a)**
Age (years)	median 64	median 66	mean 68.8		median 65.1	median 66.6	mean 64.6
	(range 44–79)	(range 50–77)	(range 49.2-75.3)		(range 52–75)	(range 45–78)	(range 52–84)
Gleason score (n) not known	10	10	-	-	3	6	5
2-4	-	-	-	-	-	-	22
5-6	-	-	-	-	-	-	60
3-6	-	-	-	-	9	11	-
2-7	55	43	-	-	-	-	-
7	-	-	-	-	26	29	-
7-10	-	-	-	-	-	-	63
8-10	33	22	-	-	9	5	-
Grade (n) G1	-	-	3	4	-	-	-
G2	-	-	13	12	-	-	-
G3	-	-	4	3	-	-	-
T stage (n) T1	3	6	2	2	-	-	43
T2	68	53	13	11	-	-	98
T3	27	16	4	6	-	-	9
T4	-	-	1	0	-	-	0
Positive surgical margins (n)	-	-	-	-	32	31	-
Positive seminal vesicle (n)	-	-	-	-	27	32	-
Nodal status (n) assessed	-	-	-	-	median 11	median 14	-
					(range 3–36)	(range 2–39)	
positive	-	-	-	-	median 2	median 2	-
					(range 1–19)	(range 1–20)	

(a). Authors reported data for baseline patient characteristics only for the group of patients with node-positive prostate cancer but not for patients randomized to early or deferred androgen suppression therapy.

**Table 3:** Study characteristics

	**RTOG-85-31**[9,10]	**Granfors et al.**[11,12]	**EST-3886**[13-15]	**EPC program**[16,17]
Design	prospective RCT (1987–1992)	prospective RCT (1986–1991)	prospective RCT (1988–1993)	prospective RCT (1995–1998)
Included participants	173 patients with lymph node-positive prostate cancer and no distant metastases at study entry; no prior AST	39 patients with lymph node-positive prostate cancer and no distant metastases at study entry; no prior AST	98 patients with lymph node-positive prostate cancer and no distant metastases at study entry; no prior AST	88 patients with lymph node-positive prostate cancer and no distant metastases at study entry; no prior AST
Local therapy	radiotherapy (65–70 Gy) with/without radical prostatectomy	radiotherapy (mean 64.9-65.2 Gy)	radical prostatectomy	radical prostatectomy (74 patients) or radiotherapy (14 patients; mean 65 Gy) (c)
Lymph node assessment	mandatory (done by lymphangiogram, computed tomography, lymphadenectomy)	mandatory (done by lymphadenectomy)	mandatory (done by lymphadenectomy)	mandatory (c) (done by lymphadenectomy, computed tomography) (d)
Intervention (early AST)	LHRH analogues (goserelin, initiated during last week of radiotherapy; 98 patients)	orchiectomy (initiated at time of local therapy; 20 patients)	LHRH analogues (goserelin)/orchiectomy (initiated at time of local therapy; 47 patients)	anti-androgen (bicalutamide 150 mg daily) (initiated at time of local therapy, 42 patients)
Control (deferred AST)	LHRH analogues (initiated at clinical progression; 75 patients) (a)	LHRH analogues/orchiectomy (initiated at clinical progression; 19 patients) (a)	LHRH analogues/orchiectomy (initiated at clinical progression; 51 patients) (a, b)	AST at investigators discretion (initiated at clinical progression; 46 patients) (a)
Follow-up	median 6.5 years for all patients, 9.5 year for survivors	median 9.3 years for all patients (14–19 years), 16.5 years for survivors	median 11.9 years	median 3 years
Definition of clinical progression	local progression: reappearance of palpable tumor after initial clearance, progression of palpable tumor (at any time), or biopsy-proven presence of carcinoma of the prostate 2 years or more after study entry. regional progression: clinical or radiographic evidence of tumor in the pelvis with or without palpable tumor in the prostate by digital examination (a)	occurrence of clinical evident local tumor growth or bone or other distant metastases (a)	evidence of recorded clinical progression or death from any cause (a)	occurrence of objective progression (confirmed by bone scan, magnetic resonance imaging, ultrasonography, or computed tomography scan) or death without progression (a)

AST, androgen suppression therapy; RCT, randomized controlled trial; Gy, Gray; LHRH, luteinising hormone-releasing hormone.

(a). Testing of prostate-specific antigen (PSA) was not used for definition of clinical progression.

(b). Use of AST was delayed if only local recurrence was suspected and physicians were advised to treat with local treatment (i.e. radiotherapy) first [13,15].

(c). Besides radical prostatectomy and radiotherapy, watchful waiting was also investigated as standard treatment. Only patients that underwent local therapy (radical prostatectomy, radiotherapy) were included in this review.

(d). Authors “assumed that most radical prostatectomy patients were assessed at surgery, suggesting that most patients with node-positive disease had a histologically confirmed nodal status” [16].

**Table 4:** Risk of bias

	**RTOG-85-31**[9,10]	**Granfors et al.**[11,12]	**EST-3886**[13-15]	**EPC program**[16,17]
random sequence generation	random number generator	not described	random number generator	random number generator
allocation concealment	central allocation	not described	central allocation	central allocation
blinding of participants/personnel	no	no	no (only pathologists were blinded)	double-blinded (placebo-controlled)
blinding of outcome assessment	unclear	unclear	unclear	unclear
incomplete outcome data	low risk (a)	low risk (a)	low risk (a)	low risk (a)
selective reporting	low risk (b)	high risk (c)	low risk (b)	low risk (b, d)
note/other bias	randomization of 977 patients but only 173 (18%) presented with lymph node-positive disease.	staging was retrospectively regraded to ensure comparable groups; initially planned for 400 patients but stopped after inclusion of 91 of which only 39 patients (43%) presented with lymph node-positive disease.	staging was retrospectively regraded to ensure comparable groups; initially planned for 220 lymph node-positive patients but stopped after inclusion of 100 of which only 98 were randomized	randomization of 8113 patients but only 150 (2%) presented lymph node-positive disease (radical prostatectomy: 74 patients, radiotherapy: 14 patients, watchful waiting: 62 patients).

(a). We found no evidence for missing outcome data for patients with node-positive prostate cancer. Additionally, survival/progression outcome data were presented by intention-to-treat.

(b). The study protocol is not available but we suggest that the published reports include all expected outcomes.

(c). One or more outcomes of interest are reported incompletely so that they cannot be entered in a meta-analysis.

(d). Authors reported data for adverse events in the subgroup of patients with node-positive prostate cancer inconsistently. However, adverse events were reported sufficiently for all patients included in the study in other reports, which were not eligible for this review.

**Table 5:** Grading the quality of evidence

**Quality assessment**							**No of patients**		**Effect**		**Quality**
**No of studies**	**Design**	**Risk of bias**	**Inconsistency**	**Indirectness**	**Imprecision**	**Other considerations**	**Early vs. deferred androgen suppression therapy**	**Control**	**Relative(95% CI)**	**Absolute**	
**Overall survival (follow-up median 6.5-11.9 years)**											
3	randomized trials	serious^1,2,3^	no serious inconsistency	no serious indirectness	serious^5,6,7,8^	none	78/165 (47.3%)	92/145 (63.4%)	HR 0.62 (0.46 to 0.84)	170 fewer per 1000 (from 64 fewer to 264 fewer)	⊕⊕ΟΟ low
**Cancer-specific survival (follow-up median 11.9 years)**											
1	randomized trials	serious^1^	no serious inconsistency	no serious indirectness	Serious^5,6^	none	7/47 (14.9%)	25/51 (49%)	HR 0.34 (0.18 to 0.64)	285 fewer per 1000 (from 140 fewer to 376 fewer)	⊕⊕ΟΟ low
**Clinical progression at 3 years (follow-up median 3-11.9 years)**											
4	randomized trials	serious^1,2,3,4^	no serious inconsistency	no serious indirectness	serious^5,6,7,8,9^	none	13/187 (7%)	44/171 (25.7%)	RR 0.29 (0.16 to 0.52)	183 fewer per 1000 (from 124 fewer to 216 fewer)	⊕⊕ΟΟ low
**Clinical progression at 9 years (follow-up median 6.5-11.9 years)**											
3	randomized trials	serious^1,2,3^	no serious inconsistency	no serious indirectness	serious^5,6,7,8^	none	43/165 (26.1%)	78/144 (54.2%)	RR 0.49 (0.36 to 0.67)	276 fewer per 1000 (from 179 fewer to 347 fewer)	⊕⊕ΟΟ low

^1^ EST-3886: Random sequence generation: Random number generator; Allocation concealment: Central allocation; Blinding of participants/personnel: No (only pathologists were blinded); Blinding of outcome assessment: Unclear; Incomplete outcome data: We found no evidence for missing outcome data for patients with node-positive prostate cancer and survival/progression outcome data were presented by intention-to-treat; Selective reporting: The study protocol is not available but we suggest that the published reports include all expected outcomes; Note: Staging was retrospectively regraded to ensure comparable groups.

^2^ Granfors et al.: Random sequence generation: Not described; Allocation concealment: Not described; Blinding of participants/personnel: No; Blinding of outcome assessment: Unclear; Incomplete outcome data: We found no evidence for missing outcome data for patients with node-positive prostate cancer and survival/progression outcome data were presented by intention-to-treat; Selective reporting: One or more outcomes of interest are reported incompletely so that they cannot be entered in a meta-analysis; Note: Staging was retrospectively regraded to ensure comparable groups.

^3^ RTOG-85-31: Random sequence generation: Random number generator; Allocation concealment: Central allocation; Blinding of participants/personnel: No; Blinding of outcome assessment: Unclear; Incomplete outcome data: We found no evidence for missing outcome data for patients with node-positive prostate cancer and survival/progression outcome data were presented by intention-to-treat; Selective reporting: The study protocol is not available but we suggest that the published reports include all expected outcomes.

^4^ EPC program: Random sequence generation: Random number generator, Allocation concealment: Central allocation; Blinding of participants/personnel: Double-blinded (placebo-controlled); Blinding of outcome assessment: Unclear; Incomplete outcome data: We found no evidence for missing outcome data for patients with node-positive prostate cancer and survival/progression outcome data were presented by intention-to-treat; Selective reporting: The study protocol is not available but we suggest that the published reports include all expected outcomes.

^5^ Heterogeneity may arise from differences in interventions (radical prostatectomy or radiotherapy) or populations (medical or surgical castration) or different lymph node assessments (lymphangiogram, computed tomography, lymphadenectomy).

^6^ EST-3886: Initially planned for 220 lymph node-positive patients but stopped after inclusion of 100 of which only 98 were randomized.

^7^ Granfors et al.: Initially planned for 400 patients but stopped after inclusion of 91 of which only 39 patients (43%) presented with lymph node-positive disease.

^8^ RTOG-85-31: Randomization of 977 patients but only 173 (18%) presented with lymph node-positive disease.

^9^ EPC program: Randomization of 8113 patients but only 150 (2%) presented lymph node-positive disease (radical prostatectomy: 74 patients, radiotherapy: 14 patients, watchful waiting: 62 patients).

### Overall survival

**Figure 2:**
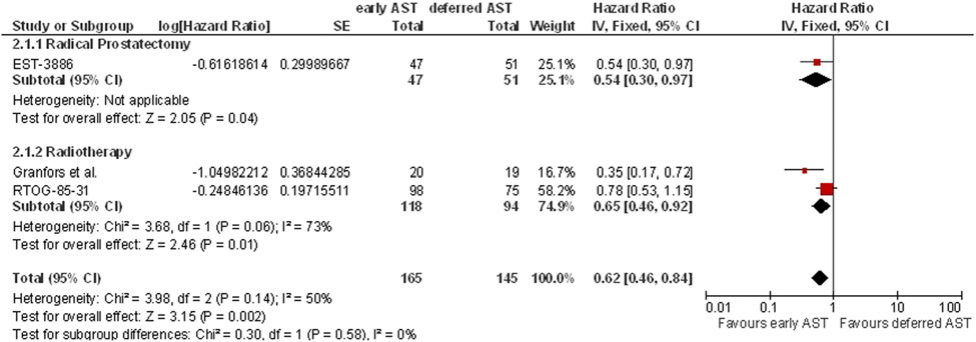
**Overall survival.** EST-3886, median follow-up 11.9 years; RTOG-85-31, median follow-up 6.5 years; Granfors 2006, follow-up 14–19 years; AST, androgen suppression therapy. (This figure should be published in the manuscript).

Three studies (RTOG-85-31, Granfors et al., EST-3886), with a combined total of 310 patients with node-positive prostate cancer, provided data on overall survival. The results demonstrated a statistically significant difference favoring early AST as compared with deferred AST after a minimum median follow-up of 6.5 years (Figure 2, HR 0.62; 95% CI 0.46-0.84). Both local treatments (radical prostatectomy and radiotherapy) revealed significant benefits of early AST (see Figure 2). However, using the random-effects model for heterogeneity (I^2^ = 73%) for overall survival with radiotherapy revealed no significant difference (HR 0.56; 95% CI 0.26-1.21; not shown). The total effect with random-effects model, however, showed still a significant benefit favoring early AST as compared with deferred AST (HR 0.57; 95% CI 0.37-0.90, not shown).

### Cancer-specific survival

**Figure 3:**
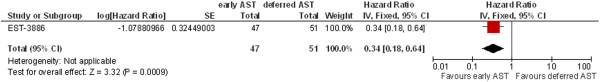
**Cancer-specific survival.** EST-3886, median follow-up 11.9 years; AST, androgen suppression therapy. (This figure should be published online only).

Two studies (Granfors et al., EST-3886) reported data on cancer-specific survival. However, we were not able to include both studies in the meta-analysis because they included insufficient amounts of detail. Granfors et al. stated only that “among lymph node positive patients there was a significantly poorer prognosis after radiotherapy alone (and AST at clinical progression; deferred AST) than after combined treatment (early AST) (p = 0.01)” [12]. EST-3886 reported data for cancer-specific survival favoring early AST as compared with deferred AST after a median follow-up of 11.9 years (Figure 3, HR 0.34; 95% CI 0.18-0.64).

### Clinical progression

**Figure 4:**
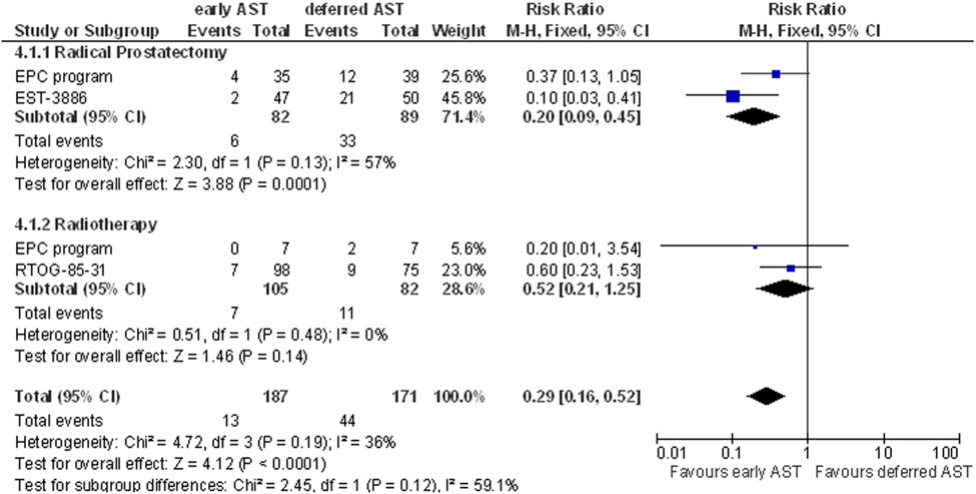
**Clinical progression at 3 years.** AST, androgen suppression therapy. (This figure should be published in the manuscript).

**Figure 5:**
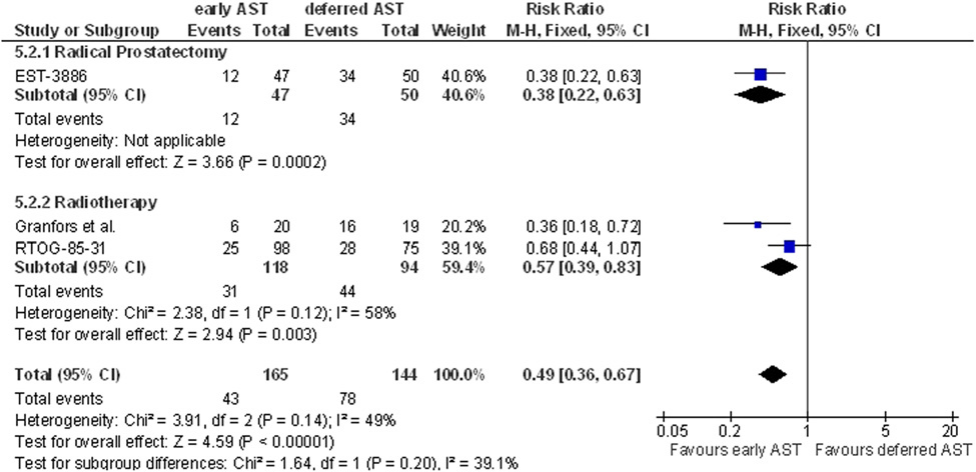
**Clinical progression at 9 years.** AST, androgen suppression therapy. (This figure should be published in the manuscript).

Four studies (RTOG-85-31, Granfors et al., EST-3886, EPC program) reported data on clinical progression. For the definitions of clinical progression used, see Table 3. Three studies were included in a meta-analysis for clinical progression at 3 years (RTOG-85-31, EST-3886, EPC program) or 9 years of follow-up (RTOG-85-31, Granfors et al., EST-3886). Pooled analysis demonstrated a statistically significant benefit for early AST as compared to deferred treatment for both time points (Figure 4, RR 0.29, 95% CI 0.16-0.52 at 3 years and Figure 5, RR 0.49, 95% CI 0.36-0.67 at 9 years, respectively). The benefit of non-steroidal antiandrogens has not been determined so far. We therefore performed a sensitivity analysis to test the robustness of results. The effect of early AST on clinical progression at 3 years remained significant after exclusion of non-steroidal antiandrogen data (EPC program) from pooled analysis (RR 0.27, 95% CI 0.13-0.56; not shown). Sensitivity analysis for clinical progression at 3 years for the subgroup treated with radical prostatectomy (I^2^ = 57%) using the random-effects model revealed a significant difference favoring early AST as compared with deferred AST (HR 0.21; 95% CI 0.06-0.78; not shown). The total effect still showed a more beneficial effect for early AST (HR 0.31; 95% CI 0.14-0.71, not shown). This was also revealed by the sensitivity analysis for clinical progression at 9 years for patients that had received radiotherapy (I^2^ = 58%). Early AST still showed a more beneficial effect than deferred AST when analyzed using a random-effects model (HR 0.52; 95% CI 0.28-0.98, not shown). The total effect was more favorable for early AST at 9 years (HR 0.47; 95% CI 0.30-0.73, not shown) 7.

### Biochemical progression

The Early Prostate Cancer Program presented data on a composite endpoint including biochemical progression (defined by the earliest occurrence of prostate specific antigen (PSA) doubling from baseline), clinical disease progression, or death in the absence of progression at 3 years’ median follow-up after radical prostatectomy in patients with node-positive prostate cancer. The authors noted a significant difference favoring early AST compared with deferred therapy that was initiated at the onset of clinical progression (HR 0.11, 95% CI 0.04-0.30) [16,17]. RTOG-85-31 reported data including PSA progression (defined as a PSA elevation of greater than 1.5 ng/ml or 4 ng/ml after 6 years’ median follow-up following radiotherapy) showing a beneficial effect of early compared to deferred AST (PSA >1.5 ng/ml: p < 0.0001 or PSA >4 ng/ml: p < 0.0001, respectively) [9]. However, RTOG-85-31 was initiated before the widespread use of PSA testing. PSA measurement was introduced later in the study. Consequently, PSA was not routinely used as a marker for biochemical disease progression in any of the studies included.

### Adverse events

Two studies (RTOG-85-31, EST-3886) involving 271 patients with node-positive prostate cancer reported data on discontinuation due to adverse events. They showed no significant differences between early and deferred AST (RR 8.44, 95% CI 0.47-150.35 (5/145 vs. 0/126), not shown). The authors did not report which outcomes led to withdrawal. The results were imprecise because the events described were rare. In addition, the studies were not placebo-controlled, and the results might therefore have been biased. Only one study with 98 patients (EST-3886) reported data on adverse events. This study demonstrated an increased occurrence of adverse events such as hematological (9/46 vs. 2/50, p = 0.02) and gastrointestinal adverse events (12/46 vs. 3/50; p < 0.01), non-specific genitourinary effects (22/46 vs. 6/50; p < 0.01), hot flashes (27/46 vs. 0/50; p < 0.001), gynecomastia (10/46 vs. 1/50; p < 0.01), and weight gain ( 8/46 vs. 1/50; p = 0.05) with early AST compared with deferred AST. The authors did not provide specific definitions of hematological, gastrointestinal, or non-specific genitourinary adverse events. The authors reported, however, that most of these events appeared with grade 1–2 severity and that AST was well tolerated [13]. No other adverse events occurred with a significantly different frequency between early and deferred AST. The data for adverse events in RTOG-85-31, the EPC program, and the study published by Granfors et al. could not be included because the authors did not report this outcome consistently for the subgroup of patients presenting with node-positive disease after local therapy. The authors of the RTOG-85-31 study reported only an increased occurrence of hot flashes and more fluid retention among patients treated with early AST as compared to deferred AST [9,10]. No study assessed the adverse events associated with skeletal changes.

## Discussion

The available evidence from RCTs might support the use of early instead of deferred AST for patients with node-positive prostate cancer following local therapy for overall survival, cancer-specific survival, and clinical progression. However, this therapy is probably associated with an increased frequency of adverse events. The quality of evidence provided by RCTs is hampered by the risk of bias.

We included data from studies assessing early versus deferred AST. The type of AST was, however, varied among the studies included. This could lead to bias because the debate concerning the equivalence of different AST therapies is still ongoing. LHRH agonists were found to be as effective as surgical castration with orchiectomy [18]. Orchiectomy is currently performed less frequently due to its irreversibility and potential negative psychological effects but is an effective therapy with which to achieve castration [18]. Current guidelines recommend that selection between these two therapy options should be made after a discussion that includes both the patients and the physicians [18]. Non-steroidal antiandrogens (high-dose bicalutamide) might be an alternative to castration for patients with locally advanced, non-metastatic disease (M0). The benefit compared with castration has not been determined [18]. However, we included the data related to bicalutamide therapy (EPC program) only for the evaluation of clinical progression at 3 years (Figure 4) and the results remained significant after exclusion of data to bicalutamide from meta-analysis. The risk of potential bias therefore remains low. Unfortunately, our data allowed no identification of outcome differences between the different AST therapy options.

We included studies assessing AST for patients that were treated with either radical prostatectomy or radiotherapy. Therefore, there might be clinical heterogeneities considering the type of local therapy that the patient received. However, we performed subgroup analyses to address potential outcome differences. Both local therapies (radical prostatectomy or radiotherapy) are often used for the treatment of localized prostate cancer. However, it must be considered that the techniques of radiotherapy and radical prostatectomy have advanced considerably since these studies were conducted. We are not able to estimate how this development can alter the outcome of patients that are currently treated. This omission should be addressed when interpreting our data.

We included data from patients with lymph node-positive disease as assessed by lymphadenectomy or imaging. In two studies (RTOG-85-31, EPC program), imaging was used to define nodal involvement associated with malignancy. Bias might occur because staging performed by any method other than lymphadenectomy might be sub-optimal. Pathological staging is much more accurate than clinical staging with computer tomography. The authors of the study conducted by the Early Prostate Cancer Program (EPC program) suggested that most patients with node-positive disease were diagnosed with radical prostatectomy and therefore had histologically confirmed nodal status [16]. However, we were unable to consider data on the extent of lymph-node involvement for all included studies. This factor is, however, known to significantly impact prognosis [19,20].

We included a total of 3 studies for the evaluation of overall survival, and all reported favorable overall survival for early AST. We were unable to identify subgroup analyses concerning node-positive patients from any of the excluded randomized controlled trials. However, non-randomized studies provide further evidence for those on node-positive prostate cancer reporting controversial results. In contrast to our results, only two observational studies demonstrated a statistically significant benefit of early AST [21,22]. Although Myers et al. reported small differences between early and deferred AST [23], results from other observational studies did not favor either of the two adjuvant therapy options [24-28]. No observational study showed a significant difference for either early or deferred AST for cancer-specific survival for patients with node-positive prostate cancer after local therapy [26-31].

Our results suggest that early AST in patients with node-positive prostate cancer after local therapy delays the progression of clinical disease. This conclusion is supported by several observational studies demonstrating an advantage for early AST as compared to deferred therapy in patients with node-positive prostate cancer [24,27,31-33]. There is also evidence from RTOG-85-31 that early AST has a beneficial effect on the incidence of metastatic disease (time from randomization to clinical or radiological evidence of disease beyond the pelvis) as compared to deferred treatment (p = 0.026) [9].

The information available on adverse events is limited, as they were not consistently reported in all studies. However, our data suggest that early AST results in an increased frequency of adverse events; this conclusion is supported by other reports [34-37]. This should be balanced against a potential improved local control [38] and a reduced occurrence of complications due to tumor progression (i.e. cord compression, ureteric obstruction, and extra-skeletal metastases) [35].

Studies included in the present review compared the administration of AST either at the time of local therapy or symptoms of clinical progression, but PSA was not routinely used as a marker for disease progression in any of the studies included. However, PSA testing currently plays an important role in the detection of prostate cancer and biochemical disease progression. In modern patient treatment, PSA testing is used to assess the risk for extraprostatic dissemination or lymph-node involvement, for example, by using nomograms [39]. This has substantially decreased the proportion of men who are upstaged with surgery. Current patient cohorts might therefore be different than those enrolled previously. It might be advisable that it is an obligation to follow high-risk patients using PSA measurement in a regular manner [22,29,40].

The application of our results is hampered by the fact that the available studies are rather outdated and included patients who might no longer represent the contemporary population. The risk of bias is also notable. However, the present study provides the best evidence on this clinically relevant topic, reporting improved survival, delayed disease progression, but also more adverse events among patients with node-positive prostate cancer after local therapy treated with early AST as compared to deferred AST. AST is challenging to clinicians for two reasons: for one, patients with advanced prostate cancer demand the highest possible quality of life and are intrigued by the thought of therapy-free intervals suggesting successful overall management. Second, the communication of hypotheses regarding the preservation of androgen dependence in earlier years left many urologists uncertain about the best use of AST and how to counsel patients. Thus, the present data offer the best currently available evidence-based guidance for clinicians. Nevertheless, new studies using modern diagnostic evaluation, biochemical testing, and standardized follow-up schedules are warranted.

## Conclusion

Only four studies could be included in this review. Available data from small RCTs with an inherent risk of bias and low quality suggest an improvement of survival, delayed disease progression, but also more adverse events among patients with node-positive prostate cancer after local therapy treated with early AST as compared to deferred AST. Large RCTs with a low risk of bias should be conducted to confirm these findings and to evaluate PSA as a trigger for the initiation of AST.

## Abbreviations

AST: Androgen suppression therapy; CI: Confidence interval; EST-3886: Study performed by the Eastern Cooperative Oncology Group; EPC program: Study performed by the Early Prostate Cancer Program; HR: Hazard ratio; LHRH: Luteinizing hormone releasing hormone; PSA: Prostate specific antigen; RCT: Randomized controlled trial; RR: Risk ratio; RTOG-85-31: Study performed by the Radiation Therapy Oncology Group.

## Competing interest

The authors declare that they have no competing interests.

## Authors’ contributions

FK and JJM designed the review. FK coordinated the review. FK, EM and BK have been involved in the search strategy and data collection for the review. FK, BK, GR and JJM did the analysis of the data. FK, BK, JJM, GA and BW interpreted the data. FK, BK, GR, EM, JJM, BW and GA wrote the review and revised the manuscript. JJM, GA and BW provided general advice on the review. FK, BK, GR, EM, BW, GA and JJM read and approved the final manuscript.

## Pre-publication history

The pre-publication history for this paper can be accessed here:

http://www.biomedcentral.com/1471-2407/13/131/prepub
